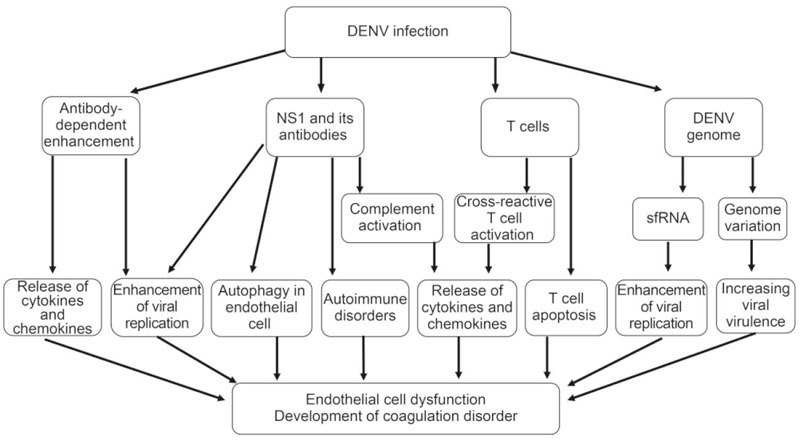# Progress towards understanding the pathogenesis of dengue hemorrhagic fever

**DOI:** 10.1007/s12250-016-3855-9

**Published:** 2016-11-14

**Authors:** Xiaojing Pang, Rudian Zhang, Gong Cheng

**Affiliations:** 1 Tsinghua-Peking Center for Life Sciences, School of Medicine, Tsinghua University, Beijing, 100084 China; 2 School of Life Science, Tsinghua University, Beijing, 100084 China

**Keywords:** DENV, dengue hemorrhagic fever (DHF), NS1, genome, antibody-dependent enhancement (ADE), T cell

## Abstract

Dengue virus (DENV) is a mosquito-borne virus belonging to the *Flaviviridae* family. There are 4 serotypes of DENV that cause human disease through transmission by mosquito vectors. DENV infection results in a broad spectrum of clinical symptoms, ranging from mild fever to dengue hemorrhagic fever (DHF), the latter of which can progress to dengue shock syndrome (DSS) and death. Researchers have made unremitting efforts over the last half-century to understand DHF pathogenesis. DHF is probably caused by multiple factors, such as virus-specific antibodies, viral antigens and host immune responses. This review summarizes the current progress of studies on DHF pathogenesis, which may provide important information for achieving effective control of dengue in the future.

## INTRODUCTION

Dengue virus (DENV) is a mosquito-borne virus belonging to the *Flaviviridae* family. There are 4 serotypes of DENV (DENV-1 through DENV-4) that cause human disease through transmission via the mosquito vectors *Aedes aegypti* and *Aedes albopictus* (Gubler, 2011). There are more than 100 dengue-epidemic countries in the world. Furthermore, approximately 2.5 billion people are at risk of dengue infection every year. It estimated 390 million dengue infections per year, of which 96 million manifest apparently any level of disease severity (Bhatt et al., 2013). In 2015, 2.35 million cases of dengue were reported in America alone, and more than ten thousand of these cases involved severe hemorrhagic-related clinical symptoms. Dengue infection remains an important threat to public health and is responsible for a large global disease burden.

Most DENV infections are asymptomatic or present a mild illness with flu-like symptoms, such as fever, headache, myalgia and decreased platelet counts and leucopenia. These symptoms are known as dengue fever (DF), which is an acute, self-limited, febrile illness. However, some DF patients develop a severe syndrome known as Dengue Hemorrhagic Fever (DHF), in which patients may display hematomas with marked thrombocytopenia or extremely low platelet counts. Even more severe clinical symptoms are produced when DENV infection develops Dengue Shock Syndrome (DSS), a disease similar to DHF that additionally includes coagulation abnormalities, increased vascular fragility, raised plasma leakage/heme concentration, loss of fluid due to capillary permeability that may progress to hypovolemic shock, and increased risk of multi-organ failure (WHO/TDR, 2009). The clinical hallmark of DHF is plasma leakage, which usually lasts for approximately 48 hours and leads to reduced circulatory volume (Srikiatkhachorn, 2009). Bleeding is common following dengue infection, however it is more frequent in DHF/DSS than in DF. Most dengue-associated deaths result from the symptoms associated with DHF/DSS (Hermann et al., 2015). There is no antiviral therapy against DENV infection. Dengvaxia, a commercial vaccine developed by *Sanofi Pasteur*, was licensed in several dengue-endemic countries (Hladish et al., 2016; Recker et al., 2016). DHF is the most life-threatening consequence of DENV infection. Previous studies have suggested that both viral and host factors might contribute to the pathogenesis of this disease, including Nonstructural protein 1 (NS1) viral antigen and its antibodies, virus variation and virulence, subgenomic RNA, antibody-dependent enhancement (ADE), and the presence of memory cross-reactive T cells. The further research on the pathogenesis of DHF can provide a new target for the prevention and control of dengue infectious diseases.

## ROLE OF DENV NONSTRUCTURAL PROTEIN-1 (NS-1) AND ITS ANTIBODIES IN DHF PATHOGENESIS

NS1, a nonstructural protein encoded by DENV, is expressed in multiple oligomeric forms and is present in a variety of cellular locations, including on intracellular membranes, at the cell surface and extracellularly as a soluble, secreted lipoparticle (Chuang et al., 2016). During acute DENV infection, secreted NS1 protein is present in patient sera at high levels (Libraty et al., 2002), which might correlate with disease severity and contribute to the pathogenesis of DHF in a host (Chuang et al., 2013; Srikiatkhachorn and Kelley, 2014). Indeed, DENV NS1 might directly bind to the surfaces of host cells to cause tissue damage (Avirutnan et al., 2007; Beatty et al., 2015; Modhiran et al., 2015). A recent study suggested that DENV NS1 elicits inflammatory cytokine production by activating macrophages and human peripheral blood mononuclear cells (PBMCs) through Toll-like receptor 4 (TLR4), thus leading to disruption of endothelial cell monolayer integrity in blood vessels (Modhiran et al., 2015). Moreover, DENV NS1 might also trigger complement activation, causing plasma leakage (Kurosu et al., 2007). During this process, soluble DENV NS1 is released from infected cells and independently activates complement factors present in the fluid phase (Nielsen, 2009). A close correlation has been found between DENV NS1 concentration and C5b-C9 complex formation. The C5b-C9 complex can stimulate robust expression of inflammatory cytokines that are associated with DHF development (Suresh et al., 2016). Recently, it has been reported that DENV NS1 may induce autophagy in the HMEC-1 human endothelial cell line as well as in mice, and that this autophagy is mediated by NS1-associated secretion of a macrophage migration inhibitory factor (MIF) (Chen et al., 2016).

In addition to the NS1 protein itself, NS1 antibodies might also contribute to DHF pathogenesis. For example, the binding of anti-NS1 antibodies to GPI-anchored NS1 proteins on cell membranes can activate cellular signal transduction pathways, leading to protein tyrosine phosphorylation, which may enhance DENV replication within infected cells (Jacobs et al., 2000). Moreover, protein phosphorylation and NF-κB activation have been observed after stimulating human HMEC-1 cells with anti-DENV NS1 antibodies. Furthermore, the expression of several cytokines and chemokines, such as IL-6, IL-8, and MCP-1, is increased after treatment of endothelial cells with anti-DENV NS1 antibodies, suggesting that DENV NS1 antibodies can stimulate the release of multiple inflammatory factors in an NF-κB-dependent manner (Lin et al., 2005). Disordered release of cytokines is considered as a major factor underlying DHF pathogenesis. Therefore, NS1 antibody-mediated activation of immune responses may play an important role in the development of Dengue-mediated thrombocytopenia and vascular leakage during the critical phase of DHF/DSS.

Recently, the induction of autoimmune disorders by anti-NS1 antibodies has also been described as a contributing factor to DHF pathogenesis. Because there is sequence homology between DENV NS1 and several self-antigens, such as plasminogen and integrin, proteins on human endothelial and platelet cells, auto-antibodies induced by NS1 may cross-react with these self-antigens (Falconar, 1997; Liu et al., 2016; Rachman et al., 2013; Chuang et al., 2016). These reactions stimulate nitric oxide (NO) expression and cell apoptosis in endothelial cells and cause cell lysis and inhibition of aggregation in platelets (Sun et al., 2007). Damage to endothelial cells and platelets may contribute to thrombocytopenia, coagulopathy and vascular leakage in DHF (Sun et al., 2007; Wan et al., 2014).

## ROLE OF DENV GENOME IN DHF PATHOGENESIS

Genetic differences among DENV genotypes are associated with differential viral virulence that may contribute to the development of the severe diseases, such as DHF and DSS (Ubol et al., 2008; Mammen et al., 2014). Some DENV genotypes have greater virulence and epidemic potential than others. This viewpoint was primarily introduced based on epidemiological observations (Gubler et al., 1981). In the American Dengue outbreak that occurred in 1981, a Southeast Asian DENV-2 genotype emerged more virulent than the indigenous American genotype (Rico-Hesse et al., 1997; Rico-Hesse et al., 1998). Indeed, evidence has indicated that a Southeastern Asian DENV-2 genotype virus replicate at higher titers than an American genotype virus in both humans and mosquitoes (Anderson and Rico-Hesse, 2006; Vasilakis

Figure 1. The mechanisms involved in DHF pathogenesis include nonstructural protein 1 (NS1) viral antigen and its antibodies, virus genome variation, sfRNA, antibody-dependent enhancement (ADE), and the presence of memory cross-reactive T cells. NS1 and its antibodies contribute to DHF pathogenesis by triggering complement activation and then eliciting inflammatory cytokine production, inducing autophagy in endothelial cell, enhancing DENV replication, inducing autoimmune disorders; DENV genome variation evolve greater virulence, and sfRNA induce the enhancement of viral replication; FcγR-mediated ADE can enhance viral replication and trigger cytokines and chemokines release; Activated DENV-specific cross-reactive T cells promote the release of cytokines and chemokines, and T cell apoptosis also contributes to DHF pathogenesis.

et al., 2007). Besides, the intra-serotype evolution of epidemic DENV strains might be another cause of increasing viral virulence. The clinical manifestations and case-fatality rates of DHF typically increase during the later periods of epidemics, a phenomenon that was observed in the Cuban epidemics of 1981 and 1997, the Townsville, Australia epidemic of 1992, and the Oaxaca, Mexico epidemic of 2005 (Guzman et al., 2000; Martina et al., 2009; Ritchie et al., 2013). An analysis of DENV genomes suggested that circulating DENV might evolve greater virulence through passage in patients during an epidemic (Chen et al., 2008). Thus, variations in the DENV genome serve to determine viral infectivity in both hosts and vectors (Ritchie et al., 2013).

Besides the genome variation, the subgenomic flavivirus RNA (sfRNA) may play a role in DENV replication in host cells, thus contributing to DHF pathogenesis. During DENV replication, a 11 kb RNA genome may be incompletely degraded from their 3' untranslated region (UTR) by host exoribonuclease to generate a 0.3 to 0.5 kb small RNA termed sfRNA (Pijlman et al., 2008; Funk et al., 2010; Chapman et al., 2014; Roby et al., 2014). sfRNA may accumulate in the infected cells to suppress the host antiviral immune responses, such as type I interferon signaling (Manokaran et al., 2015; Chang et al., 2013). sfRNA may also facilitate DENV replication through altering host mRNA stability (Moon et al., 2012; Schnettler et al., 2012), thereby acting as a player in DENV immune evasion and DHF pathogenesis.

## ROLE OF ANTIBODY-DEPENDENT ENHANCEMENT (ADE) IN DHF PATHOGENESIS

Epidemiological evidence has shown that increased risk for DHF/DSS is associated with a secondary infection from a different DENV serotype than that causing the initial infection (Guzman et al., 2013). Data gathered from Cuba, Hawaii and Thailand have shown that individuals with circulating DENV 1 antibodies are at an enhanced risk for acquiring DHF during subsequent DENV 2 or DENV 3 infections (Alvarez et al., 2006). Furthermore, an infant infected with a DENV serotype that is different from the serotype that its infected mother is at an increased risk for DHF/DSS (Halstead et al., 2002). Based on assessments of hospital patients, the incidence of DHF/DSS during primary dengue infections was 11 to

12 per 1000 compared with 118 to 208 per 1000 during secondary dengue infections (Guzman et al., 2013). The risk of developing DHF during a secondary infection is at least 10-fold greater than the risk of developing the condition during a primary infection (Srikiatkhachorn, 2009). This increased infection rate is caused by antibodies induced from the first infection and has been termed antibody-dependent enhancement (Halstead, 1970). Indeed, the ADE phenomenon has been repeatedly demonstrated in DENV infections of cell cultures and animals. When DENV is incubated with diluted anti-DENV sera or DENV-specific antibodies, viral replication in human monocytes and some cell lines is significantly promoted. Passive immunization with DENV antibodies/antisera prior to virus inoculation in rhesus monkeys enhanced viral replication more than 100-fold over control animals (Goncalvez et al., 2007; Sasaki et al., 2013; Chaichana et al., 2014; Martinez gomez et al., 2016). Nonetheless, not all cases of DHF/DSS are associated with ADE. Evidence from a phase 3 clinical trial of a Dengue vaccine (*Sanofi Pasteur*) found no evidence of ADE generation in the study population (Sabchareon et al., 2012). Although ADE has been primarily described in relation to DENV infection, the phenomenon has also been associated with several other viral species based on *in vitro* enhancements of infection (Takada et al., 2003; Martina et al., 2009; Chan-Hui and Swiderek, 2016).

Previous studies have demonstrated that ADE are directed toward anti-E antibodies and anti-prM antibodies. Although more studies have been designed to evaluate the role of E protein, prM have attracted more attention since 2010, when it was first proposed that anti-prM antibodies involved in the ADE phenomenon (Dejnirattisai et al., 2010). Several groups have shown that anti-prM MAbs exhibit ADE properties in cell culture and in animal models of DENV infection (Smith et al., 2012; Luo et al., 2013). Thus, anti-prM antibodies are likely to play an important role in the pathogenesis of DHF in humans.

The mechanism of ADE remains only partially understood. Fcγ receptor (FcγR)-mediated uptake of virion-antibody complexes into permissive cells may be one explanation for the enhancement of DENV infection (Sato et al., 2015). FcγR is a multi-subunit complex that is distributed on the surfaces of many types of immune cells, such as dendritic cells, macrophages and mast cells, and recognizes the Fc regions of immunoglobulins. During DENV infection, antibodies with infection-enhancing activity are generated and subsequently form virion-antibody complexes. These complexes can rapidly internalize into FcγR-bearing cells via interactions with FcγR, which results in a higher number of infected cells in the presence versus the absence of antibodies (Halstead, 1982). Moreover, previous studies have also shown that FcγR-mediated DENV infection might enhance viral replication through suppression of intracellular antiviral innate immune responses, including interferon-mediated antiviral responses, and enhancement of IL-10 production in DENV-infected cells. The suppression of host immune responses may promote the production of high numbers of infectious DENV virions (Ubol and Halstead, 2010).

The clinical manifestations of DHF include endothelial cell damage, enhancement of vascular permeability, plasma leakage and liver injury (Halstead and Cohen, 2015). However, these manifestations may be not necessarily a direct result of cell death induced by DENV replication because viral load peaks during the febrile phase and rapidly declines by the time hemorrhagic fever and plasma leakage develop. In addition to enhancing viral entry, FcγR-mediated ADE directly triggers robust release of cytokines from mast cells and other immune cells, which may mediate vascular endothelial cell dysfunction and increase vascular permeability. The release of cytokines is considered a key factor that contributes to DHF pathogenesis (King et al., 2002; Brown et al., 2011).

## ROLE OF T CELLS IN DHF PATHOGENESIS

The increases in cytokine production and CD8+ T cell activation that have been observed in severe dengue patients imply that cross-reactive T cells have a role in mediating the pathogenesis of DHF (Mathew and Rothman, 2008). Memory T-cells from the primary infection were activated by a heterologous virus during second infection, and showed highly cross-reactivity and low affinity leading to inefficiency at killing infected cells and clearing the newly infective virus serotype. On the contrary, cross-reactive T cell contribute to DHF occurrence by substantial immunopathology. DHF patients have been reported to demonstrate enhanced circulation of cytokines and chemokines, IFN-γ, IL-2 and TNF-α produced by activated DENV-specific cross-reactive T cells, compared to DF patients (Bozza et al., 2008; Malavige et al., 2012; Kurane et al., 2011), suggesting a positive correlation between cross-reactive T cell activation and the severity of dengue-associated disease. Furthermore, T cell apoptosis in DHF patients contributes to the severity of dengue-associated disease (Jaiyen et al., 2009; Midgley et al., 2011; Malavige et al., 2012). A previous study reported that the immunosuppressive cytokine IL-10 could lead to T cell apoptosis in patients with acute dengue infection. IL-10 concentrations are elevated in the sera of patients with severe dengue (Mathew and Rothman, 2008). IL-10 blockade has been significantly associated with reduced T cell apoptosis in acute DENV infections. In agreement, reduced T cell numbers have been found in DHF patients compared to DF patients (Green et al., 1999), suggesting that T cell apoptosis reduces viral clearance and consequently results in an impaired antiviral response, leading to severe clinical dengue diseases.

## CONCLUSION

Clinicians and scientists have increasingly focused on understanding the pathogenesis of DHF from several perspectives; however, the mechanisms underlying this pathogenesis are not yet fully elucidated. Further studies are urgently required to improve our understanding of DHF pathogenesis, which is important for developing effective therapeutics for and vaccine strategies against DENV infection.

## COMPLIANCE WITH ETHICS GUIDELINES

The authors declare that they have no conflict of interests. This article does not contain any studies with human or animal subjects performed by any of the authors.

## OPEN ACCESS

This article is distributed under the terms of the Creative Commons Attribution 4.0 International License (http://creative commons.org/licenses/by/4.0/), which permits unrestricted use, distribution, and reproduction in any medium, provided you give appropriate credit to the original author(s) and the source, provide a link to the Creative Commons license, and indicate if changes were made.
